# Improving the Visibility and Accessibility of Physical Health Information in a Forensic Medium-Secure Inpatient Unit

**DOI:** 10.1192/bjo.2024.365

**Published:** 2024-08-01

**Authors:** Rijul Bohra, David Kelsey, Jon FitzGerald

**Affiliations:** 1 Oxleas NHS Foundation Trust, London, United Kingdom; 2 South London and Maudsley NHS Foundation Trust, London, United Kingdom

## Abstract

**Aims:**

To improve the visibility and accessibility of secure inpatients’ physical health needs by measuring staff satisfaction levels towards physical health information and monitoring.

**Methods:**

Through purposive sampling we conducted a five-point Likert Scale on members of the multidisciplinary team (MDT) on one medium-secure forensic ward, within Oxleas NHS Foundation Trust, which provides 124 forensic inpatient beds to southeast London. We collated physical health data from across electronic patient records to create a single-point-of-access workspace on Microsoft OneNote, accessible to all members of the MDT contemporaneously, comprising past medical history, psychotropics requiring close monitoring (e.g. lithium, clozapine, valproate), vital signs, weight, bloodwork, electrocardiogram findings, hospital appointments/results and cancer screening.

We re-sampled members of the ward MDT after the workspace had been created and implemented.

**Results:**

Nine members of the multidisciplinary team were sampled before and after the OneNote workspace was implemented.

- Pre-intervention, 56% disagreed that they were confident in quickly viewing recent investigation results. Post-intervention, 99% of users agreed/strongly agreed, with no negative responses.
- Pre-intervention, 67% disagreed or strongly disagreed that they were confident in knowing what physical health appointments were scheduled. Post-intervention, 100% of respondents agreed/strongly agreed.
- Pre-intervention, 78% disagreed or strongly disagreed that they were happy with the availability of past medical history information. Post-intervention, this increased to 99% agreed/strongly agreed.
- Pre-intervention, 89% disagreed/strongly disagreed that they knew where to see patients on psychotropics requiring close monitoring. Post-intervention, this increased to 100% strongly agreed.
- Pre-intervention, 66% disagreed/strongly disagreed being able to see single-point, up-to-date physical health information, at baseline. This increased to 99% agreed/strongly agreed post-intervention.

Overall, 89% agreed/strongly agreed the workspace would allow them to better understand the physical health and monitoring needs of patients, whilst 78% agreed/strongly agreed it allows for more effective work across wards/sites in the Oxleas forensic directorate.

**Conclusion:**

Physical health information is often overlooked in secure inpatient settings. Due to the limitations of the electronic patient record, it can be difficult to find relevant physical health information quickly. This can lead to dissatisfaction and a lack of confidence in the MDT, as shown in the baseline data.

After the Microsoft OneNote dashboard was introduced, there was a marked improvement in staff confidence, happiness, and awareness of physical health requirements for each patient.

Further data needs to be collected to assess for sustainability of these improvements. We intend to expand the scope of this system across the secure inpatient units in the Trust.

